# Supplementary material for: Understanding high pressure molecular hydrogen with a hierarchical machine-learned potential
Source: Nat Commun. 2020 Oct 6;11:5014. doi: 10.1038/s41467-020-18788-9 (PMC7538439; doi:10.1038/s41467-020-18788-9)
Supplement: Supplementary file 1 — Supplementary Information [file 41467_2020_18788_MOESM1_ESM.pdf]

Supplementary Information: "Understanding high pressure molecular hydrogen with a hierarchical machine-learned potential"

Hongxiang Zong[1,2], Heather Wiebe[1] and Graeme J. Ackland[1]

September 8, 2020

1. Centre for Science at Extreme Conditions and School of Physics and Astronomy, University of Edinburgh, Edinburgh, EH9 3FD, UK

2. State Key Laboratory for Mechanical Behavior of Materials, Xi'an Jiaotong University, Xi'an, Shanxi 710049, China

Supplementary Note 1

Our choice of the PBE function comes from its status as the *de facto* standard in studies of molecular systems and high pressure hydrogen. PBE has been criticised for overstabilising the metallic phases, however in this work we only consider molecular phases, so this is not a concern. In our 2017 paper: "The role of van der Waals and exchange interactions in high-pressure solid hydrogen S Azadi, GJ Ackland PCCP 19, 21829" we showed that the various functionals including vdW corrections give just as large a spread of results as those without. To further illustrate this point, we recalculated the fitted configurations using the using the vdW-DF proposed by Dion et al (implemented as optPBE-vdW in VASP). As shown in Supplementary Figure 1, the vdW term makes very little difference apart from a systematic shift in the energy due mainly to using the free-atom as a reference state.

Supplementary Note 2

Here, we show an example of how the database is updated by the iterative scheme. The earlier MD simulations at 100 GPa and 50 K get a wrong phase structure, as shown in Supplementary Figure 2a. To optimize the HMLP, we add the DFT calculations of these unexpected structures to the database, and refitting the potential gives rise to structures of Supplementary Figure 2b. Upon further iterations, we successfully got the $P2_1/c$-24 structure when the MD simulations are re-run with the re-fitted potential (Supplementary Figure 2c).

Supplementary Note 3

In order to simulate large systems, the total energy is expressed as a linear combination of the sum of local energy contributions from all the atoms. In this scenario, each atomic

energy contribution depends only on its local environment, which is represented by a feature space vector or fingerprint so as to make the problem more amenable to a machine-learning representation.

Our fingerprint includes sums of pair potentials for non-bonded component, a Harmonic + Morse potential for the covalent intramolecular interaction, and a orientation-dependent interaction.

We describe the short-ranged Coulomb and van der Waals potentials using pairwise functions to create the fingerprint. These are built using Gaussians with a smooth cutoff in the form

$$V_i^k = \sum \exp(-|r_{ij}/\eta_k|^2) f_{cut}(r_{ij}) \tag{1}$$

which are combined with damped sinusoidal functions in the form

$$V_i^k = \sum \sin(kr_{ij}) \exp(-r_{ij}/\eta_k) f_{cut}(r_{ij}) \tag{2}$$

where $\mathbf{r}_{ij}$ is the distance between atom i and j,and k is assigned as integers from the 1 to 8, η_k=$\eta_0 1.28^{k-1}$ with $\eta_0 = 0.6$.

$f_{cut}(r_{ij}) = 0.5[1 + \cos(\pi r_{ij}/R_c)]$ is a damping function for atoms within the cutoff distance $R_c = 6.5$ Å .

The orientation-dependent interaction has two parts: atom-molecule interaction and molecule-molecule interactions. The atom-molecule interaction is captured by a term with the form

$$V_i^n = \sum_{ml} g_n(\cos\theta_{mil}) \exp(-|(r_{im} - r_{il})/\eta_k|^2) f_{cut}(r_{im}) f_{cut}(r_{il})$$

where m, l label atoms in the same molecule. We choose $g_n(\cos\theta) = 1.0$, $\cos\theta$, $3\cos^2\theta - 1$ and $5\cos^3\theta - 3\cos\theta$ while other parameters are the same as before.

As for the molecule-molecule interactions, we first build 96 possible rotationally-invariant function forms that are collected from Ref. S2 as well as their combination. In order to improve the computational efficiency and reduce the generalization error, a sequential feature selection (SFS) algorithm is adopted to select the best 16 function forms, which are listed in Supplementary Table 2. The details of the SFS algorithm is illustrated in Supplementary Note 4.

The next step is to map the fingerprints to non-bonded component of the corresponding energies and classical Hellmann-Feynman forces derived from standard density functional theory. Here, we have adopted the kernel ridge regression (KRR) method, capable of handling both linear and nonlinear relationships. In the present work, the local energy corresponding to pairwise interaction is given by a linear combination of kernel functions:

$$E_i^{pairwise} = \sum_t W_t K(V_i, V_t) + b_0 \tag{3}$$

Here, K is a linear kernel function of the form $K(x, y) = x{\cdot}y$, whereas W_t and b_0 denote the weighting coefficient and a constant obtained from the fitting procedure, respectively. t labels each reference atomic environment and V_t is its corresponding fingerprint vector.

Finally, we consider the orientation-dependent component. These are fitted to the residuals once covalent and pairwise interactions are subtracted from the DFT energies. A similar function form to Eq. (3) is used to fit, that is

$$E_i^{orient} = \sum_t W_t K(V_i, V_t) + b_1 \tag{4}$$

Here, W_t and b_1 denote the weighting coefficient and a constant. Unlike the pairwise interaction, here an rbf kernel function of the form $K(x, y) = \exp(-\sigma|x - y|^2))$ is used, where σ is length-scale parameter ($\sigma = 0.001$ in this work for the sake of fitting precision).

All the parameters are determined during the training process, with the help of 5-fold cross-validation and L2 regularization methods. More details of the cross-validation and L2 regularization can be found in the textbook of machine learning [S3-S5]. All the fitted parameters are provided in the supplementary code package. {Eventually, the cross-validation gives rise to a low mean absolute error, indicating a high fidelity between our ML prediction and the DFT calculations where the inherent error of the DFT data is not included in the error evaluation. It also implies a good transferability of the HMLP among Phases I, II, III and liquid below 160 GPa. But this strategy should be easily extended to the description of phase transition in higher pressure region.

Our HMLP is further assessed by illustrating how the hierarchical model can achieve increasing accuracy. Correlation plots of atomic force and potential energy already make this clear (Supplementary Figure 3): using the covalent term only (violet squares) or the combination of covalent and nonbonding terms (dark blue squares), there is a certain degree of correlation between the DFT and ML prediction, but with much scatter while adding orientation-dependent term dramatically improve the fidelity of our interatomic potential (cyan circles).

Supplementary Note 4

The details of the selection algorithm is addressed in Supplementary Figure 4. It is outlined in pseudo code below:

Input: the set of all 96 features, $Y = \{y_1, y_2, ..., y_{96}\}$

Output: a subset of features, $X_k = \{x_j | j = 1, 2, ..., k; \quad x_j \in Y\}$

Initialization: $X_0 = Y, \quad k = 96$

Step 1 (Conditional Exclusion):

1) Use greedy search algorithms to find three features from our feature subset, X_k, and remove them to if they can improve the fitting precision; 2) $k = k - 3$; 3) Go to Step 2;

Step 2 (Conditional Inclusion):

Search for two features (from the removed ones) that improve the performance of the ML potential if they are added back to the feature subset, X_k; 2) $k = k + 2$; 3) Go to Step 1;

Termination: Stop when the number of features in X_k equals 16.

References

S1. Dion, M., Rydberg, H., Schröder, E., Langreth, D. C., and Lundqvist, B. I., Van der Waals Density Functional for General Geometries. *Phys. Rev. Lett.*, **92**, 246401 (2004).

S2. Koide,A., Angle-dependent intermolecular forces between linear molecules. *J. Phys. B: Atom. Molec. Phys.*, **11**, 633 (1978).

S3. Kung, S. Y. Kernel Methods and Machine Learning. (Cambridge University Press, Cambridge, England, 2014)

S4. Raschka, S. Python Machine Learning. (Packt Publishing, Birmingham, 2015)
S5. Rostamizadeh, A., Talwalkar, A., Mohri, M. Foundations of Machine Learning (the MIT Press, London, 2012)

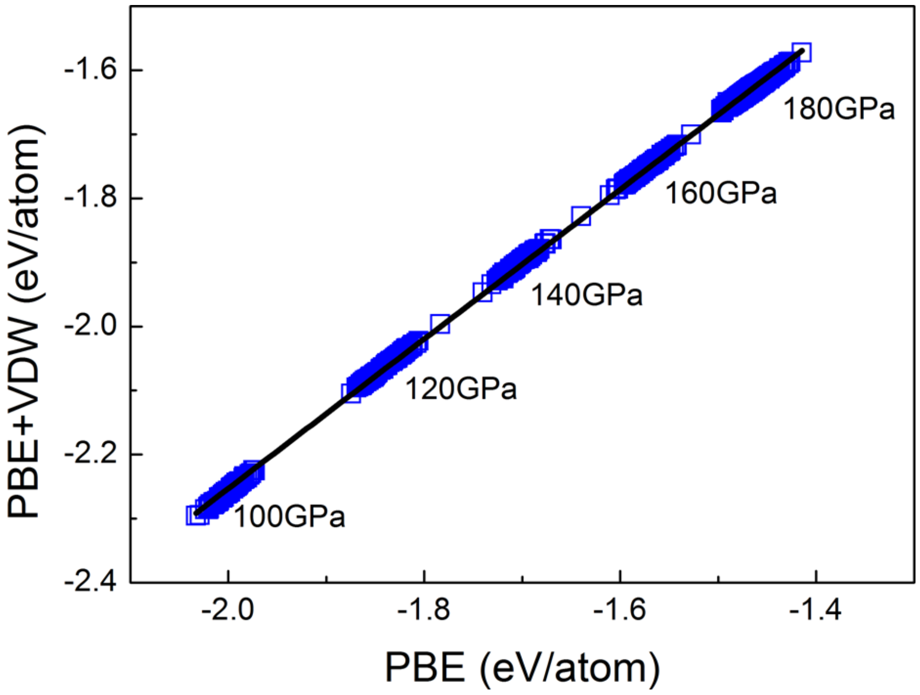

Supplementary Figure 1. Comparison of energies of fitted configurations, from MD at various pressures, with and without vdW corrections.

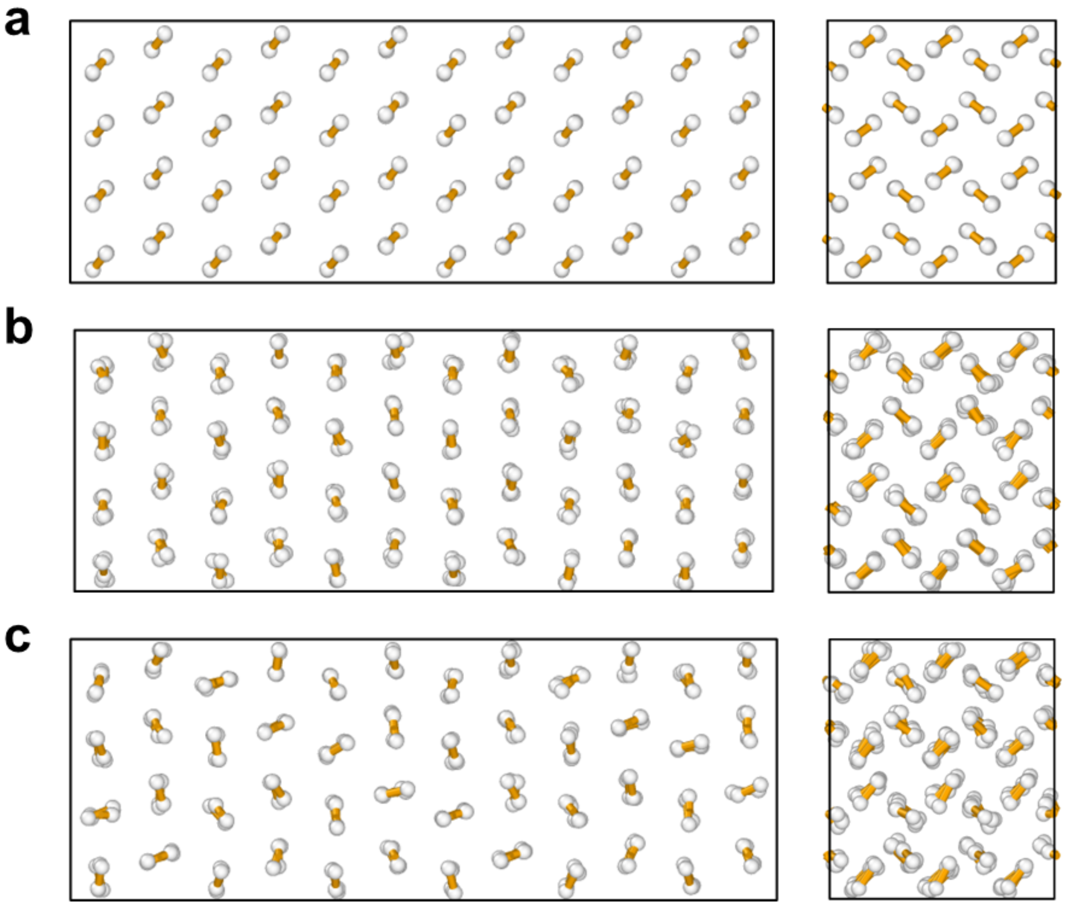

Supplementary Figure 2. Structures from MD simulation tests in our iterative process a and **b** shows unexpected structures from the MD simulation with earlier potential at pressure of 100GPa and temperature of 50 K. **c.** $P2_1/c$-24 structure from the MD simulation with updated potential.

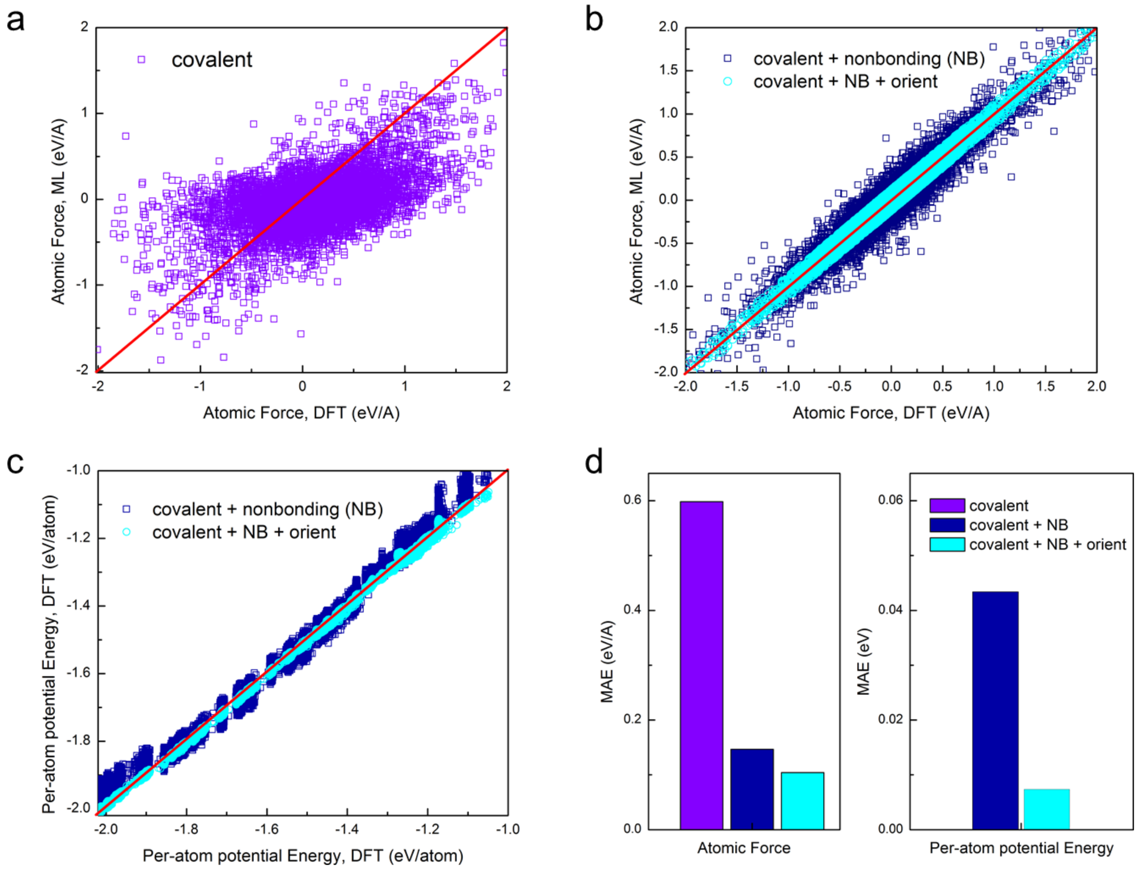

Supplementary Figure 3. Performance of our ML potential after each hierarchical step, comparing DFT to fit a. Force comparison from fitting only the covalent term, with no intermolecular interactions. The DFT data used to fit this term is $(F_1^{\mu_{12}} - F_2^{\mu_{12}})/2$, where $F_1^{\mu_{12}}$ is the component of the atomic force projected down the molecular axis. **b.** Comparing atomic forces after fitting inte-rmolecular nonbonding terms (NB) and further improvement from adding orientation-dependent contribution. **c.** Scatter plots for potential energy of various configurations with or without the orientation-dependent contribution. A perfect correlation with the DFT values would correspond to the red lines. **d.** Comparison of the mean absolute error (MAE) after each different hierarchical step.

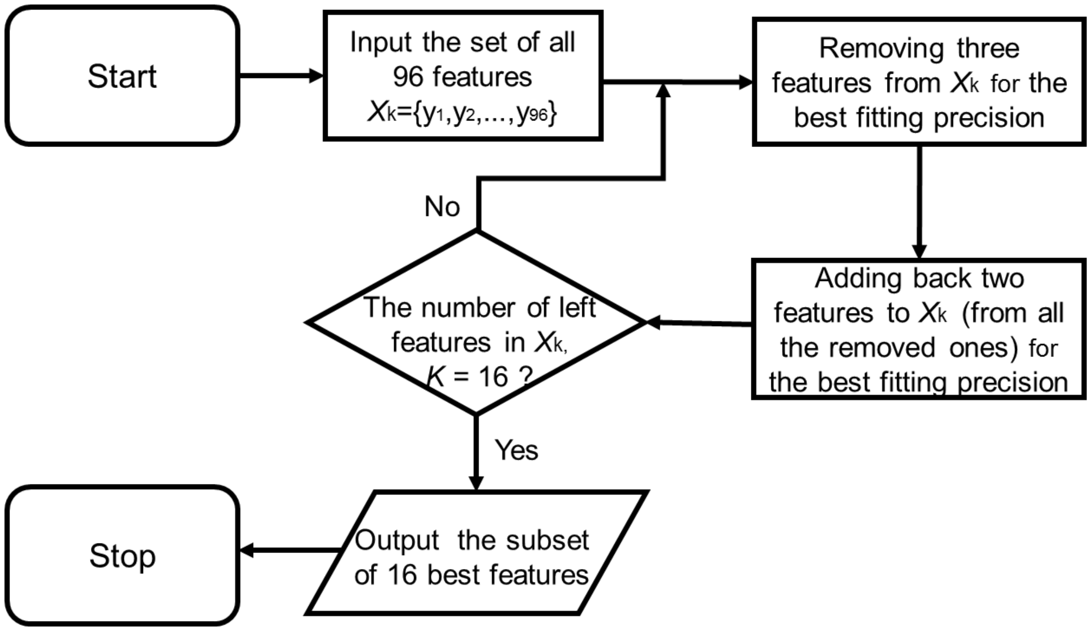

Supplementary Figure 4. Flow chart of the sequential feature selection (SFS) algorithm used in the present work. The SFS algorithm helps us select 16 best features from the 96 candidates. The details of the SFS algorithm is illustrated in Supplementary Note 3.

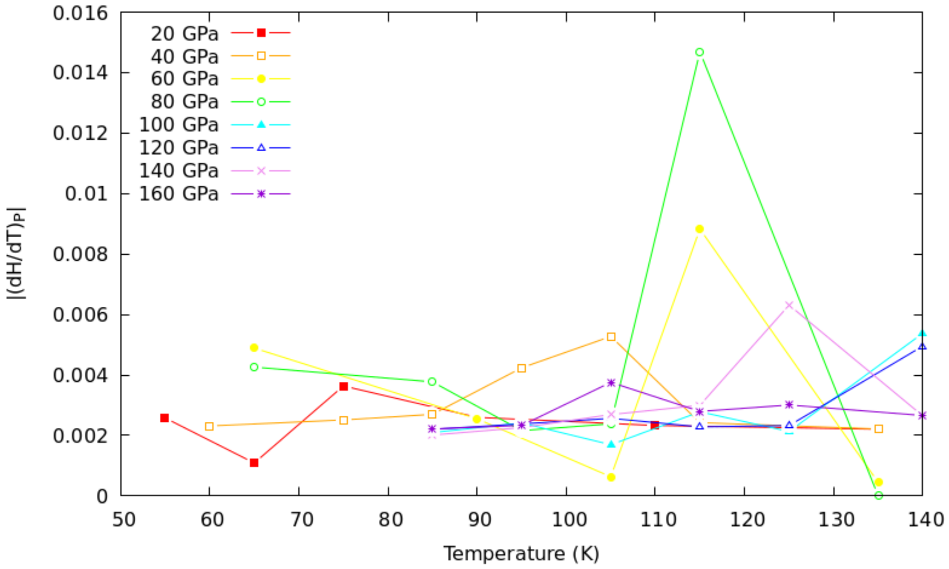

Supplementary Figure 5. Phase I-II transition from heat capacity. In addition to $\langle O \rangle$, the I-II phase boundary was located by identification of peaks in the constant pressure heat capacity, calculated from the MD trajectories as $\left(\frac{\partial H}{\partial T}\right)_P$. The locations of these peaks agree with the discontinuous changes in $\langle O \rangle$ illustrated in the main text.

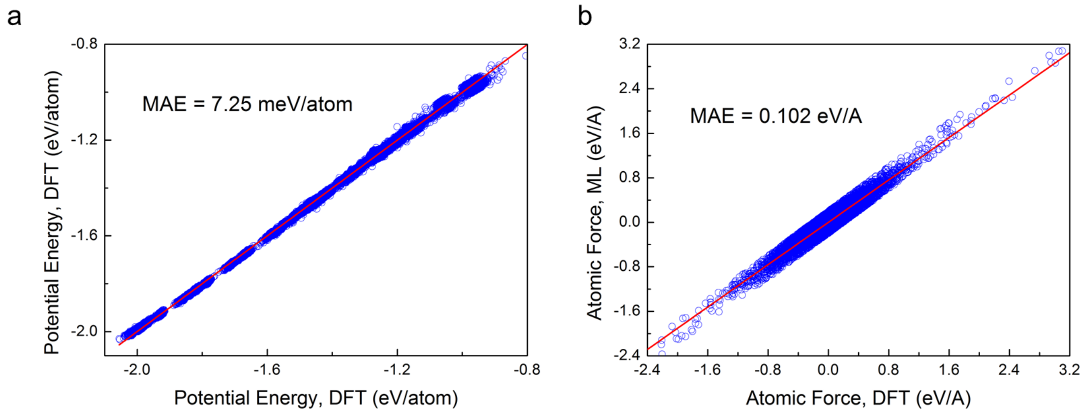

Supplementary Figure 6. Performance of our ML potential compared with the DFT AIMD references. a. Per-atom potential energy of all configurations in the training dataset. **b.** Atomic force for randomly selected 300 configurations in the training dataset. A perfect correlation with the DFT values would correspond to the red lines. MAE represents mean absolute error.

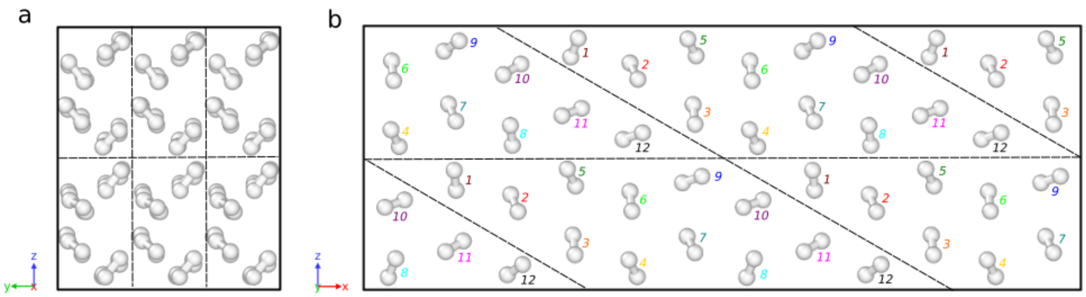

Supplementary Figure 7. Typical structure of $P2_1/c$-24 for solid H_2. The $P2_1/c$-24 phase II basis used in this work shown in **a** top-down and **b** side views. Shown here is a small 2x3x2 supercell, for clarity's sake. The unit cells are denoted by the dashed lines. The twelve molecules that form the basis in each unit cell are numbered in the side view.

Supplementary Table 1. Key parameters for the H-H bonding models.		
Harmonic	$K(eV/\mathring{A})$	33.86
	$r_e(\mathring{A})$	0.730
Morse	$D_e(eV/\mathring{A})$	13.611
	$\alpha(\mathring{A}^{-1})$	2.4352
	$r_e(\mathring{A})$	0.7365
For the Harmonic term, the bonding energy is given by $0.5K \cdot (r - r_e)^2$, the bonding energy in Morse model is expressed as $D_e \cdot (e^{-2\alpha(r-r_e)} - 2e^{-\alpha(r-r_e)})$		

Supplementary Table 2. Fingerprinting the molecule-molecule interactions.	
e^{-2R}	$(3cos^2\phi - 1)R^{-3}$
R^{-1}	$cos^2\theta_1 cos^2\theta_2 R^{-3}$
$r_1 r_2(1 - 3cos^2\phi)R^{-1}$	$cos\phi cos\theta_1 cos\theta_2 R^{-3}$
$r_1 r_2 R^{-3}$	$(1 - 3cos^2\theta_1)R^{-5}$
$cos^2\phi cos^2\theta_1 R^{-3}$	$r_1 r_2(1 - 3cos^2\phi)R^{-5}$
$cos^2\phi cos^2\theta_2 R^{-3}$	$(1 - 3cos^2\theta_2)R^{-5}$
$cos^2\theta_1 R^{-3}$	$(35cos^4\theta_1 - 30cos^2\theta_1 + 3)R^{-5}$
$(3(cos^2\phi + cos^2\theta_1 + cos^2\theta_2) - 9cos\phi cos\theta_1 cos\theta_2 - 2)R^{-5}$	
$(1 - 5(cos^2\theta_1 + cos^2\theta_2 + 3cos^2\theta_1 cos^2\theta_2) + 2(cos\phi - 5cos\theta_1 cos\theta_2)^2)R^{-5}$	
R is the distance between molecular centers. r_1 and r_2 are the bond length of the H_2 molecules.The orientation of the molecules with respect to the R axis is specified by angles θ_1 and θ_2. ϕ is the relative twist angle between two molecules.	
