# Supplementary material for: Understanding high pressure molecular hydrogen with a hierarchical machine-learned potential
Source: Nat Commun. 2020 Oct 6;11:5014. doi: 10.1038/s41467-020-18788-9 (PMC7538439; doi:10.1038/s41467-020-18788-9)
Supplement: Supplementary file 3 — Description of Additional Supplementary Files [file 41467_2020_18788_MOESM3_ESM.docx]

Supplementary Data 1

File Name: HMLP-plugin-for-LAMMPS.zip

Description: The Supplementary Data is a compressed file which includes our HMLP plugin code for the MD simulation code LAMMPS, a readme file which explains how to compile and run the code, as well as an example of input files.
